# A Comparative Study of Automated Machine Learning Platforms for Exercise Anthropometry-Based Typology Analysis: Performance Evaluation of AWS SageMaker, GCP VertexAI, and MS Azure

**DOI:** 10.3390/bioengineering10080891

**Published:** 2023-07-27

**Authors:** Wansuk Choi, Taeseok Choi, Seoyoon Heo

**Affiliations:** 1 Department of Physical Therapy, International University of Korea, Jinju 17731, Republic of Korea; wschoi@iuk.ac.kr; 2 Department of Medical Performance Center (MPC), Sejong Sports Medicine and Performance Hospital, Seoul 05006, Republic of Korea; 3 Department of Occupational Therapy, College of Medical and Health Sciences, Kyungbok University, Namyangju 12051, Republic of Korea

**Keywords:** artificial intelligence, AutoML, kinematic analysis, cloud-based ML models

## Abstract

The increasing prevalence of machine learning (ML) and automated machine learning (AutoML) applications across diverse industries necessitates rigorous comparative evaluations of their predictive accuracies under various computational environments. The purpose of this research was to compare and analyze the predictive accuracy of several machine learning algorithms, including RNNs, LSTMs, GRUs, XGBoost, and LightGBM, when implemented on different platforms such as Google Colab Pro, AWS SageMaker, GCP Vertex AI, and MS Azure. The predictive performance of each model within its respective environment was assessed using performance metrics such as accuracy, precision, recall, F1-score, and log loss. All algorithms were trained on the same dataset and implemented on their specified platforms to ensure consistent comparisons. The dataset used in this study comprised fitness images, encompassing 41 exercise types and totaling 6 million samples. These images were acquired from AI-hub, and joint coordinate values (x, y, z) were extracted utilizing the Mediapipe library. The extracted values were then stored in a CSV format. Among the ML algorithms, LSTM demonstrated the highest performance, achieving an accuracy of 73.75%, precision of 74.55%, recall of 73.68%, F1-score of 73.11%, and a log loss of 0.71. Conversely, among the AutoML algorithms, XGBoost performed exceptionally well on AWS SageMaker, boasting an accuracy of 99.6%, precision of 99.8%, recall of 99.2%, F1-score of 99.5%, and a log loss of 0.014. On the other hand, LightGBM exhibited the poorest performance on MS Azure, achieving an accuracy of 84.2%, precision of 82.2%, recall of 81.8%, F1-score of 81.5%, and a log loss of 1.176. The unnamed algorithm implemented on GCP Vertex AI showcased relatively favorable results, with an accuracy of 89.9%, precision of 94.2%, recall of 88.4%, F1-score of 91.2%, and a log loss of 0.268. Despite LightGBM’s lackluster performance on MS Azure, the GRU implemented in Google Colab Pro displayed encouraging results, yielding an accuracy of 88.2%, precision of 88.5%, recall of 88.1%, F1-score of 88.4%, and a log loss of 0.44. Overall, this study revealed significant variations in performance across different algorithms and platforms. Particularly, AWS SageMaker’s implementation of XGBoost outperformed other configurations, highlighting the importance of carefully considering the choice of algorithm and computational environment in predictive tasks. To gain a comprehensive understanding of the factors contributing to these performance discrepancies, further investigations are recommended.

## 1. Introduction

Kinematic morphological analysis has developed in parallel with the advancement of various methods. These methods include classical tape measures, graduated mirrors, and wearable sensors that directly attach to the body and transmit signals to a terminal [1]. Wearable sensor technology has been employed in studies of gait, balance, and range of motion. Recently, the use of high-performance automatic machine learning (AutoML) technology or deep learning methods has increased for posture estimation and kinematic analysis of human body motion [2]. Human posture estimation (HPE) is a longstanding research area in computer vision that involves estimation of the body [3,4]. This technology utilizes human motion data collected from sensors, such as cameras and markers, to recognize behavior through interpretation. A crucial aspect of HPE is feature extraction from the input signal, and various models have been developed for this purpose, including skeleton-based, contour-based, and volume-based models. The skeleton-based model, which connects key points corresponding to human joints, is the most widely used [5]. The posture could be represented by key-point (x, y, z) coordinates in the skeleton model, and these coordinates could be used for posture recognition [6]. One method of estimating fitness posture is by using deep learning to classify the type of exercise or determine the correctness of motion, by extracting key points from a sequence of images using libraries such as Mediapipe [7].

The popularity of home and personal training has led to the development of deep learning models for fitness posture estimation. Early research in this field used recurrent neural networks (RNNs) as the primary sequence model [8]. However, the vanishing gradient problem of RNNs was addressed by using long short-term memory (LSTM) with added gates [9]. Subsequently, the simpler gated recurrent unit (GRU) was used [10]. However, both LSTM and GRU models have difficulty capturing all information in a fixed-length vector for long sentences, and this issue was addressed by the attention model [11]. The transformer model, which uses weight vectors for each vector, has been recently introduced and used [12].

Auto machine learning (AutoML) has not been widely applied to fitness image learning, despite its ability to simplify the machine learning model development process. AutoML eliminates the need for data refinement, feature targeting, model selection, and hyperparameter tuning [13]. However, it is crucial to execute the omitted process in order to achieve optimal performance when utilizing an existing RNN-based model for posture estimation. AutoML also incurs costs for cloud file storage and resource usage. However, compared to traditional deep learning methods, AutoML is more economical and reduces the time for model development. Vertex AI, Amazon Web Services Amazon SageMaker, and Microsoft Azure are representative examples of AutoML [14], and are well-known for their performance in image classification in supervised learning. It is expected that these tools will perform similarly in the training of large-scale fitness sequence images [15].

In conclusion, this study aims to compare the performance of existing RNN models in classifying fitness posture images using representative AutoML tools, Vertex AI, SageMaker, and Azure. The study aims to demonstrate the advantages of using AutoML for posture estimation and to improve the performance of existing RNN models.

## 2. Methods

### 2.1. Dataset Preparation and Definition of Each Exercise

The information of “Fitness Posture Image AI Data” was obtained from AI-hub which is AI integrated platform operated by the Korea Intelligence Society Agency [16] and its data is state-managed that is permitted to be distributed to researchers or the general public for public interest purposes.The dataset consists of 200,000 15 s long video clips captured from five multi-view cameras at 360 degrees, providing a total of 6,397,721 video images in FHD resolution (1920 × 1080) and MOV file format. The data structure is organized based on the exercise state, type, posture, name, model, camera, and label serial number.

In this dataset, different postures of the same exercise are defined as different states of the exercise. For instance, the exercise of push-ups could become different states based on five sub-items such as the neutral spine, elbow angle, chest movement, hand position, and head flexion/extension. The total number of defined exercise states in the dataset is 816, with 32 states for 17 full-body workouts, 16 states for nine barbell/dumbbell exercises, and 8 states for 14 furniture-based exercises.

The dataset includes 41 exercise names and different numbers of models, with 17 full-body workouts (43%), 16 barbell/dumbbell exercises (24% barbell, 19% dumbbell), and 8 furniture-based exercises (15%). The five postures include standing (20), supine (6), prone (3), with bench (4), and with furniture (8). The label serial number is assigned to each image in the order of motion, with each exercise repeated 4–5 times within 32 frames. The total frame count for each exercise ranges from 50,000 to 260,000, calculated based on the number of motion states, models, cameras (5), and one cycle frame (32).

The dataset features a 76% male to 24% female ratio, with the largest portion of subjects (41%) being 27–29 years old. The subjects are representative of 20% of the average Korean physique and have agreed to the use of their portraits. The subjects have 2–5 years of exercise experience, with an average of 70 men and women. To improve the quality of the dataset, the same motion was repeated by more than five people, with an average of 48 people being directly photographed for each exercise. Three to five correct postures were defined for each exercise, and incorrect scenarios were created based on the correct postures.

### 2.2. Skeleton Data Extraction

**Figure 1:**
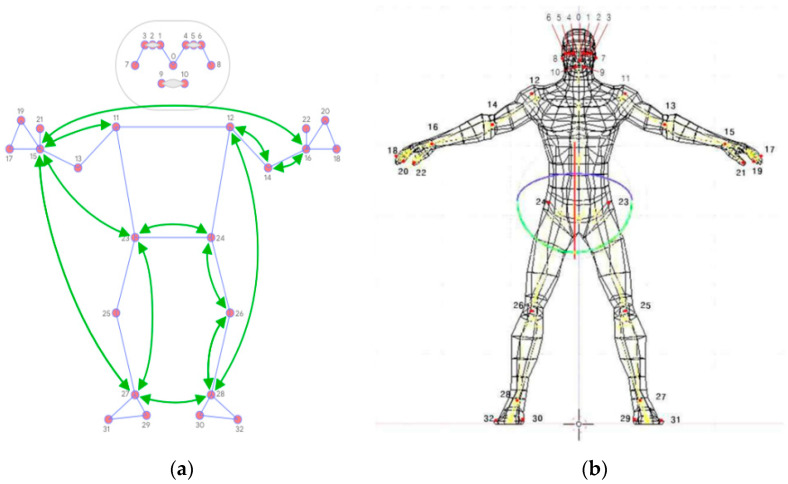
Main pairwise distances used for the pose feature vector; (**a**) a simplified version of the human body model that can be used to represent the kinematics that the camera typically recognizes, with each joint represented by a red dot and the arrow indicating the direction of the joint moment that can be recognized; (**b**) in general, the authors of this paper have modified and created an evolved human body model that can be used to represent camera-perceived kinematics in a more complex and three-dimensional form. Each joint is labeled with a generalized number, and arrows indicate the direction of the joint moment that can be used to recognize motion.

In this study, the AI-hub dataset of fitness posture images was analyzed to extract 33 keypoints per image. The Mediapipe library was utilized in conjunction with the BlazePose machine learning model to extract the joint coordinates. The Mediapipe library detects body parts in images and tracks the keypoints, represented as three-dimensional coordinates normalized to the image height and width. The processing was conducted in “static image mode” with a minimum detection confidence of 0.5 and a model complexity of 2 for accurate key-point values (Figure 1).

The images were classified into 41 different exercises and organized into separate folders (01 Standing Side Crunch; 02 Standing Knee Up; 03 Burpee Test; 04 Step Forward Dynamic Lunge; 05 Step Backward Dynamic Lunge; 06Side Lunge; 07 Cross Lunge; 08 Good morning; 09 Front Raise; 10 Upright Row; 11 Barbell Stiff Deadlift; 12 Barbell Row; 13 Dumbbell Bent-Over-Row; 14 Barbell Deadlift; 15 Barbell Squat; 16 Barbell Lunge; 17 Overhead Press; 18 Side Lateral Raise; 19 Barbell Curl; 20 Dumbbell Curl; 21 Lying Leg Raise; 22 Crunch; 23 Bicycle Crunch; 24 Scissor Cross; 25 Hip Thrust; 26 Frank; 27 Push Up; 28 Knee Push Up; 29 Y-Exercise; 30 Dumbbell Chest Fly; 31 Dumbbell Incline Chest Fly; 32 Dumbbell Pullover; 33 Lying Triceps Extension; 34 Dips; 35 Pull Up; 36 Lat Pull Down; 37 Face Pull; 38 Hanging Leg Raise; 39 Cable Crunch; 40 Cable Push Down; 41 Rowing Machine). Python code was written to extract the joint coordinates using the Mediapipe library and BlazePose. The extracted data were saved in a CSV file, and missing data were not accounted for. The exercises were labeled from 0 to 40 and the data were combined into a single file for analysis in MATLAB, due to Excel’s limitation of one million rows.

### 2.3. AutoML Processing

A posture estimation model using fitness images was built for use in clinical workflows by physical therapists, occupational therapists, or fitness majors. However, the construction of such a model requires advanced knowledge in various areas such as coding languages (e.g., Python and MATLAB), image recognition computer vision technology, data processing, and predictive analysis. These skills could be challenging to acquire, particularly through graduate professionals or in collaboration with the engineering community. These concepts are illustrated in Figure 1, which has been modified by the authors to make it easier to understand.

The computational demands of the machine learning model also pose a significant challenge. The classification of six million fitness images, even with a high-performance computer, could take several days to tens of days without a GPU or with a low-spec GPU. Additionally, the design and development of machine learning models could be complex, particularly in regard to feature engineering and data preprocessing. Feature engineering, which involves cleaning data, removing noise and context, blocking items, and segmentation, could greatly impact the quality of the model. Furthermore, the complexity of posture could result in missing data, making postural estimation difficult.

To address these challenges, functional engineering and kinematics laboratories need training and collaboration in the field of machine learning. AutoML has the potential to simplify the engineering process and overcome these considerations to some extent.

**Figure 2:**
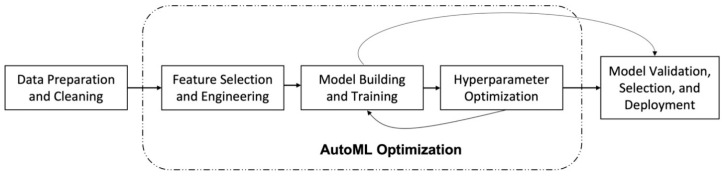
Common components of machine learning problem pipelines. The first step in the process is data preparation, which involves loading and organizing the data, as well as applying transformations, normalizations, or encodings. The next step is feature selection, which also includes the process of feature engineering, the use of domain knowledge to create new features that could enhance the performance of the machine learning models. Finally, an iterative approach is utilized to build, train, optimize, validate, and select the appropriate machine learning algorithm for the given problem.

The classification learning was performed using Amazon SageMaker for AWS, Vertex AI for GCP, and Azure Machine Learning for MS Azure. To use these platforms, users must register and pay for services. The process of developing a fitness image classification model involves uploading a dataset to the cloud storage, importing images into the platform UI, and setting up detailed parameters before training the model. All data must be labeled according to the category to be classified. The platform will then automatically perform training, validation, and testing using an 8:1:1 ratio, respectively, and identify the best-performing algorithms in the training data collection. The platform employs a validation and inference process to evaluate the performance of the model (Figure 2).

### 2.4. Amazon Web Service (AWS): SageMaker

The following steps outline the process for using Amazon SageMaker for machine learning tasks:

1. Sign up for Amazon Web Services (AWS) and navigate to Amazon SageMaker.
2. Select “Create Notebook Instance” and configure the notebook instance.
3. In “Permissions and Encryption”, select “Create new role” and choose the bucket in the “Create IAM role”.
4. Once the notebook instance is created and its status changes to “InService”, open Jupyter and create a new conda_python3 file.
5. Access the data storage section by clicking “S3” under “Storage” and create a new bucket.
6. Upload the data file to the newly created bucket and folder.
7. Register your name in the control panel by clicking “Add User”.
8. Start the machine learning process by clicking “Start App” and selecting the desired machine learning tasks and components.
9. Import the data file from Amazon S3 and set the experimental name and target.
10. Set the objective metric and runtime and review the settings before clicking “Create Experiment”.

**Figure 3:**
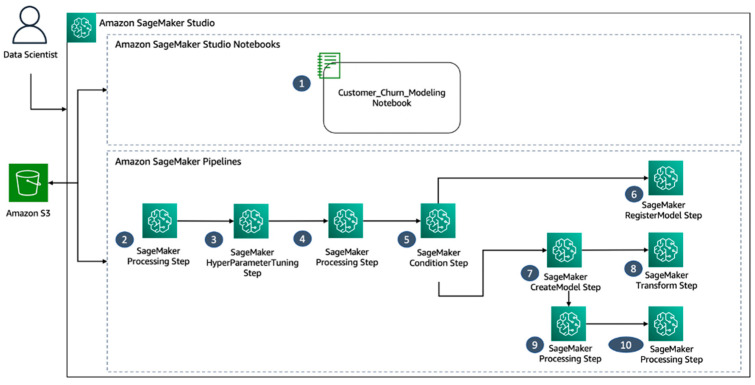
The high-level architecture of the data science workflow in Amazon SageMaker Studio. In this solution, the integrated development environment (IDE) provided by SageMaker Studio serves as the entry point for rapid experimentation. Studio provides a comprehensive platform for managing the entire end-to-end pipeline experience, eliminating the need for using the AWS Management Console for workflow management. For additional information on managing pipelines within Studio, refer to the guide “View, Track, and Execute SageMaker Pipelines in SageMaker Studio”.

This process will initiate the artificial intelligence learning process, including data preprocessing, feature engineering, model training, and the generation of an explainability and insights report (Figure 3).

The workflow consists of the following steps:

1. “Development of a Customer Churn Model Utilizing Studio Notebooks within an Integrated Development Environment (IDE)”
2. “Data Preprocessing for Feature Construction and Division into Training, Validation, and Test Datasets”
3. “Hyperparameter Tuning via the SageMaker XGBoost Framework to Determine the Optimal Model Based on AUC Score”
4. “Evaluation of the Optimal Model Utilizing the Test Dataset”
5. “Determination of Adherence to a Specified AUC Threshold for Model Selection”
6. “Registration of the Trained Churn Model in the SageMaker Model Registry”
7. “Creation of a SageMaker Model Using Artifacts from the Optimal Model”
8. “Batch Transformation of the Dataset via the Created Model”
9. “Creation of a Configuration File for Model Explainability and Bias Reports, Including Columns to Check for 10. Bias and Baseline Values for SHAPley Plot Generation”
10. “Use of Clarify with the Configuration File for Generation of Model Explainability and Bias Reports” [17].

### 2.5. Google Cloud Platform (GCP): Vertex AI

1. After signing up for the Google Cloud Platform and entering payment information, the Console was accessed.
2. The project was named in Google Cloud Storage—Bucket and the CSV file for learning was uploaded in the upper left menu.
3. The Google Cloud Artificial Intelligence—Vertex AI—Dataset location was then accessed.
4. AutoML Tables could create supervised machine learning models from tabular data with a variety of data types and problem types such as binary classification, multi-class classification, and regression.
5. Vertex AI is the successor to AutoML Tables and offers a unified API and new features. Hence, this study was conducted in the Vertex menu.
6. In the Vertex AI—Dataset, the “Dataset Name” was recorded, and the “Table Type—Regression/Classification” was selected from the “Data Type and Target Selection” menu.
7. The region was then selected from the “Region” menu and “Create” was clicked.
8. In the Dataset section, “Add Data to Dataset—Select Data Source—Select CSV File from Cloud Storage” was clicked.
9. The file path was browsed to specify the file that had already been uploaded to the cloud, and “Continue” was clicked.
10. The maximum file size allowed is 10 GB, which was sufficient for the 4 GB of data.
11. The “Generate Statistics” button was clicked on the right to check the outline of the data to be learned.
12. To train a new model, the “Learn new model” button in the upper right corner was clicked, and “Train new model” was chosen.
13. In the “Train new model—learning method” submenu, “Classification” was selected under “Objective”, and “AutoML” was chosen under “Model Training Method”.
14. “Continue” was clicked, and the “Train new model—model details” screen was reached.
15. “New model learning” was selected, and the “Target column” was set to “class”.
16. In the data partitioning menu, 80% of the data was allocated for training, 10% for validation, and 10% for testing.
17. “Continue” was clicked, and the “Train new model—learning options” menu was reached.
18. In “Customize Transformation Options”, “Automatic Option” was selected among the options of auto, categorical, text, timestamp, and number.
19. “Continue” was clicked, and the “Train new model—computing and pricing” menu was reached.
20. “7” was entered as the maximum node time for model training in the “Budget” menu, and “Enable Early Stopping” was selected.
21. The learning process was started by clicking the “Start learning” button at the bottom of the “Train new model” menu.

The pipeline components in the workflow are as follows:

1. Feature-Transform-Engine: This component performs feature extraction. For further information, refer to the Feature Transform Engine.
2. Split-Materialized-Data: This component splits the materialized data into three sets, namely, the training set, the evaluation set, and the test set.

Input: Materialized data

Output: Materialized_train_split, Materialized_eval_split, Materialized_test_split

**Figure 4:**
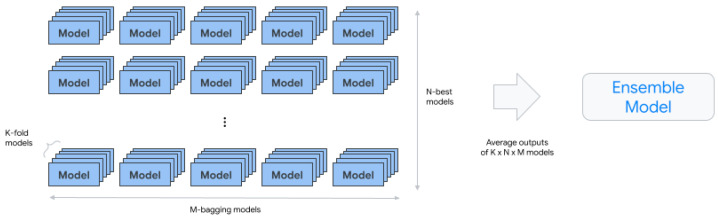
AutoML-Tabular-Ensemble.

**Figure 5:**
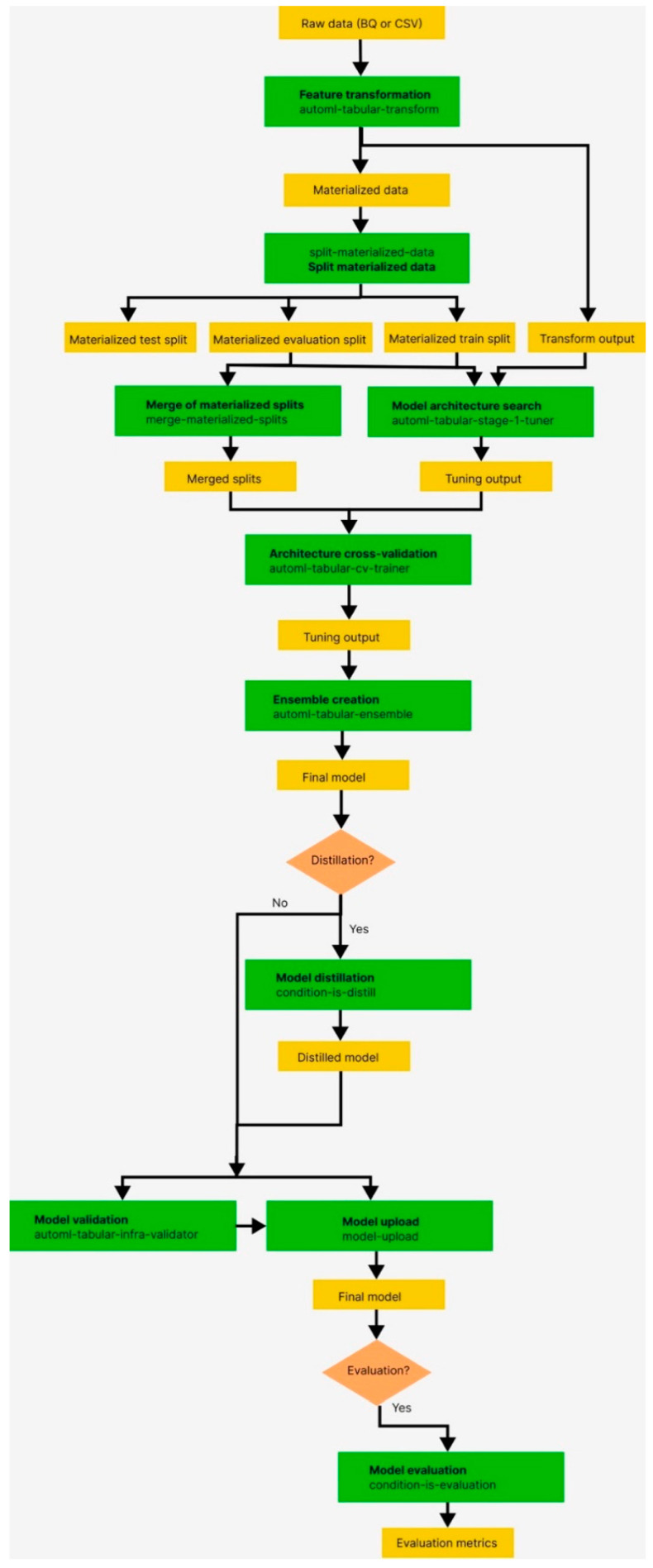
End-to-end AutoML in Vertex AI pipelines. The ‘Tabular Workflow for End-to-End AutoML’ of Vertex AI is a serverless service provided by Vertex AI, which allows automation and monitoring of machine learning and data preparation tasks. This service operates on top of the Kubeflow Pipeline, dividing the machine learning workflow into stages to perform specific tasks at each stage, such as data splitting, data type conversion, and model training. These stages are instances of pipeline components, each characterized by inputs, outputs, and a container image. The input of a stage can be set directly or derived from the output of another stage within the same pipeline. As a result, a Directed Acyclic Graph (DAG) is created that defines the workflow of the pipeline and the relationships between each stage.

3. Merge-Materialized-Splits: This component merges the materialized evaluation and training splits.
4. AutoML-Tabular-Stage-1-Tuner: This component retrieves the model architecture and optimizes the hyperparameters.
  - The architecture is defined by a set of hyperparameters, including the model type (such as neural networks or boosted trees) and model parameters.
  - A model is trained for each considered architecture.
5. AutoML-Tabular-CV-Trainer: This component cross-validates the architecture by training the model on different parts of the input data.
  - The best-performing architecture from the previous steps is considered.
  - Approximately 10 of the best architectures are selected, with the exact number determined by the learning budget.
6. AutoML-Tabular-Ensemble: These component ensembles the architecture best suited for generating the final model.
  - The following diagram represents K-fold cross-validation using bagging.
7. Condition-Is-Discip (Optional): This component generates smaller ensemble models to optimize prediction latency and cost.
8. AutoML-Tabular-Infra-Validator: This component validates the trained model to ensure its validity.
9. Model-Upload: This component is responsible for uploading the validated model.
10. Condition-Is-Evaluation (Optional): This component calculates evaluation metrics based on the Materialized_test_split output [18] (Figure 4 and Figure 5).

### 2.6. Microsoft (MS); Azure

To utilize Azure (https://azure.microsoft.com/en-us/) (accessed on 15 January 2023), proceed as follows:

1. Click on “Get Started” and log in.
2. Register account information and set relevant details in the “Create Subscription” section, including name, account, billing profile, bill, and plan.
3. Select the “Quick Start Center” and choose “Get started with data analytics, machine learning, and intelligence”.
4. From the options, select “Quickly Build and Deploy Models with Azure Machine Learning” and initiate the creation process.
5. In the “Create Machine Learning Workspace” section, enter the relevant details, including the region and storage account.
6. Review the contents and initiate the deployment process. Once the deployment is complete, access the resource by clicking the “Go to resource” button.
7. In the “Work with models in Azure Machine Learning Studio” section, click the “Start Studio” button and select “Automated ML Tasks” under the “New” menu.
8. Initiate the process to create a new automated ML job by uploading the relevant file.
9. In the task configuration, set the experiment name and select the subject column. Select the appropriate compute type, either “Compute Cluster” or “Compute Instance”.
10. Determine the virtual machine settings, including the computer name, minimum and maximum number of nodes, and idle time before scaling down.
11. Start the learning process by specifying the action and settings, such as checking classification and using deep learning.
12. Configure additional settings and view the Featurization Settings.
13. In “Validation and Testing”, set the validation and testing percentage of the data to 10.
14. Upon entering the relevant details and clicking the “Finish” button, the learning process will commence after loading for approximately 20 s. It is expected to take approximately 2 h to complete the supervised learning process for a file size of 4.48 GB.

**Figure 6:**
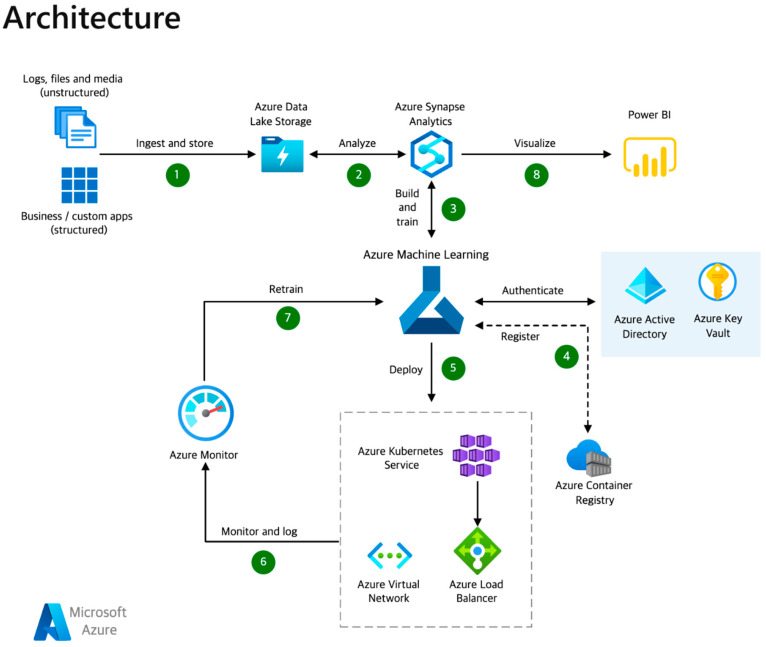
Azure Machine Learning architecture. This architecture illustrates the components utilized in building, deploying, and managing top-notch models with Azure Machine Learning, an end-to-end ML lifecycle service.

Dataflow:

1. Structured, unstructured, and semi-structured data, such as logs, files, and media, should be gathered into Azure Data Lake Storage Gen2 for efficient dataflow management.
2. Datasets should be cleaned, transformed, and analyzed using Apache Spark in Azure Synapse Analytics for optimal data processing.
3. Machine learning models should be constructed and trained in Azure Machine Learning for effective model development.
4. Access and authentication for data and the ML workspace should be managed with Azure Active Directory and Azure Key Vault, while container management should be overseen with Azure Container Registry.
5. Machine learning models should be deployed to a container with Azure Kubernetes Services, while ensuring deployment security and management through Azure VNets and Azure Load Balancer.
6. Model performance should be monitored through log metrics and monitoring with Azure Monitor for effective model evaluation.
7. Models should be continuously retrained as necessary in Azure Machine Learning for optimal model performance.
8. Data outputs should be visualized through Power BI for efficient data visualization [19] (Figure 6).

## 3. Classification Evaluation Metrics

The field of artificial intelligence (AI) is constantly evolving, driven by the rapid advancement of deep learning technology. Researchers are exploring various ways to improve the performance of AI models, such as adjusting factors such as the number of layers, epochs, and learning rate to enhance the accuracy of posture estimation in sequences. However, such adjustments require significant investments of time, effort, and monetary resources for hardware operations. Therefore, an efficient solution is to use automated machine learning (AutoML) on a cloud platform to optimize hyperparameters.

Additionally, it is crucial to evaluate the performance of the learned model to demonstrate its efficacy. Evaluation metrics for models differ based on whether they are regression, classification, unsupervised models, etc. For classifiers, evaluation metrics include accuracy, precision, recall, ROC-AUC, log loss, and F1-score, and are mainly evaluated based on the confusion matrix (TP, TN, FP, FN, etc.). It is essential to understand the terms in the confusion matrix and their definitions:

- True (T): The prediction is accurate.
- False (F): The prediction is inaccurate.
- Positive (P): The model predicts a positive outcome.
- Negative (N): The model predicts a negative outcome.
- True positive (TP): The model correctly predicted a positive outcome, and the actual answer was indeed positive.
- True negative (TN): The model correctly predicted a negative outcome, and the actual answer was indeed negative.
- False positive (FP): The model incorrectly predicted a positive outcome, but the actual answer was negative.
- False negative (FN): The model incorrectly predicted a negative outcome, but the actual answer was positive.
- False Positive Rate (FPR): The false positive rate provides the proportion of incorrect predictions in the positive class.

Accuracy is a measure of how well a model classifies positive and negative instances, and a higher value indicates higher prediction accuracy. Precision measures the accuracy of positive predictions, with a higher precision indicating fewer false positive predictions. Recall measures the percentage of correct items that were successfully predicted by the model, and a higher recall means fewer false negatives or missing predictions. The F1-score is the harmonic mean of precision and recall and is used to evaluate the performance of a model when the class distribution is uneven. A higher F1-score indicates better performance, and the closer the score is to 1, the better the performance. The F1-score is preferred over the arithmetic mean because the number of subclasses for each exercise is uneven. The ROC curve visualizes the relationship between recall and fall-out (false positive rate) based on different threshold values. A model with a high recall and low fall-out is considered a good model. The ROC curve is difficult to compare using a numerical value, so the area under the curve (AUC) is used instead. Log loss measures the log average of the target class probabilities and ranges from 0 to infinity. A smaller log loss value indicates a higher quality model.

$$(Accuracy)=\frac{TP+TN}{TP+FN+FP+TN}$$

$$(Precision)=\frac{TP}{TP+FP}$$

$$(Recall)=\frac{TP}{TP+FN}$$

$$\left(F1-score\right)=2\times \frac{1}{\frac{1}{Precision}+\frac{1}{Recall}}=2\times \frac{Precision\times Recall}{Precision+Recall}$$

$$FPR=\frac{FP}{TN+FP}$$

log loss=−1N∑i=1N(log⁡(Pi))

## 4. Experimental Results

### 4.1. Model Evaluation

The authors conducted an automated machine learning (AutoML) evaluation using platforms from three companies to classify 41 fitness images. Each platform showed varying performance results and used different performance indicators. Specifically, AWS did not provide performance results for PR AUC and ROC AUC, GCP did not provide F1 binary, AUC, balanced accuracy, and algorithm, and MS Azure did not provide F1 binary, PR AUC, and ROC AUC. However, the commonly provided performance indicators, such as F1-score, log loss, recall, precision, and accuracy, are sufficient to discuss the results. Among the platforms, AWS had the highest values for accuracy, precision, recall, and F1-score, followed by GCP and MS Azure. Log loss was highest in MS Azure, followed by GCP and AWS.

For learning algorithms, AWS SageMaker had XGBoost and KNN linear learners, with XGBoost being selected as the best performing algorithm. GCP had linear learner, wide and deep, TabNet, and XGBoost, but their use was not disclosed in the results. MS Azure supports various classification algorithms, such as logistic regression, light GBM, gradient boosting, decision trees, etc., and used LightGBM in this study. AWS allowed for the evaluation of up to 40 algorithms at the same time, and the best performing algorithm was presented.

The data were divided into 8:1:1 for training: validation: testing, and the confidence threshold was set at 0.5 for all platforms, except in GCP where it could be freely specified. Each prediction was assigned a confidence score, and a confidence threshold determined if a prediction was positive. A higher confidence threshold leads to higher precision but lower recall, and vice versa. The learning time for AWS was less than 40 min, for MS Azure it was about 2 h and 30 min, and for GCP it was about 8 h.

### 4.2. Model Comparison

The primary aim of this research paper is to conduct a comparative evaluation of the performance of various machine learning models when utilized in diverse environments. The models under investigation include recurrent neural networks (RNNs), long short-term memory (LSTM), gated recurrent unit (GRU), XGBoost, and LightGBM. The evaluation encompasses several performance metrics, namely, accuracy, precision, recall, F1-score, and log loss.

Recurrent neural networks (RNNs), LSTMs, and GRUs were all subjected to testing within the Google Colab Pro environment. The RNNs achieved an accuracy of 72.95%, precision of 70.46%, recall of 69.28%, F1-score of 68.57%, and log loss of 0.85. Surpassing the RNNs’ performance, the LSTMs displayed an accuracy of 73.75%, precision of 74.55%, recall of 73.68%, F1-score of 73.11%, and log loss of 0.71. Notably, the GRU model exhibited an accuracy of 73.26%, precision of 74.55%, recall of 73.33%, F1-score of 73.18%, and log loss of 0.74. It is worth mentioning that despite the different model architectures, both LSTM and GRU models demonstrated identical precision values of 74.55%, suggesting a similar ability to accurately identify true positive cases.

In the evaluation of AutoML models, three distinct environments were utilized: AWS SageMaker, GCP Vertex AI, and Microsoft Azure. Notably, the XGBoost model implemented on AWS SageMaker outperformed other models, exhibiting outstanding accuracy (99.6%), precision (99.8%), recall (99.2%), F1-score (99.5%), and an impressively low log loss of 0.014. These results indicate a reduced level of uncertainty and superior predictive performance. The model’s exceptional performance can be attributed to its proficiency in effectively handling both binary and multiclass classification problems by constructing an ensemble of weak prediction models, typically in the form of decision trees.

The model deployed on GCP Vertex AI, whose specific type remains undisclosed (“hidden”), demonstrated a respectable accuracy of 89.90%, precision of 94.20%, recall of 88.40%, F1-score of 91.20%, and log loss of 0.268. Conversely, on Microsoft Azure, the LightGBM model achieved an accuracy of 84.20%, precision of 82.20%, recall of 81.80%, F1-score of 81.50%, and log loss of 1.176. While these models performed less effectively when compared to XGBoost on SageMaker, they still exhibit considerable potential for specific applications.

The performance of each model significantly varies based on the specific environment and model architecture. The employed performance metrics provide invaluable insights into the models’ capabilities to make accurate predictions and correctly identify positive instances in the data. Furthermore, these metrics prove beneficial when evaluating and comparing the performance of different models, thereby aiding the decision-making process in selecting the most suitable model for specific applications (Table 1).

## 5. Discussion

The primary objective of this study was to conduct a critical assessment of various machine learning algorithms concerning their performance in terms of accuracy, precision, recall, F1-score, and log loss. The analysis employed RNNs on ML Colab Pro, as well as Auto ML on AWS SageMaker, GCP Vertex AI, and MS Azure, serving as the primary tools for investigation. Within the ML Colab Pro environment, the implementation of the GRU algorithm yielded results of 73.26% accuracy, 74.55% precision, 73.33% recall, 73.18% F1-score, and 0.74 log loss. Notably, GRU is widely acknowledged for its effectiveness in handling time series data [20].

The authors utilized an automated deep learning model, implemented via AutoML, to classify various exercise types in fitness photos. The study was conducted on cloud platforms offered by AWS, Google, and Microsoft, with the aim of comparing the mean performance of our AutoML models to existing models. The results showed that the mean performance of our models was comparable or not far behind the performance of existing models, with AWS SageMaker demonstrating the highest accuracy, precision, recall, and F1-score.

The classification performance of our model was evaluated using accuracy, precision, and practicality, with the results measured using a confusion matrix (TP, TN, FP, FN, etc.) and 10% of the data for each class image that was not used for AI training. The fitness dataset used in the study is comprised of 50,000 to 260,000 images per class, leading to a large deviation. To mitigate this, the F1-score was also presented, as evaluating the model solely based on accuracy could lead to bias. The mean precision was 92.07, indicating a 92% accuracy, and the mean recall was 89.80, indicating that the model correctly classified about 90% of all fitness images in the test data. The mean F1-score was 90.73, indicating a 91% ability to accurately classify. The log loss was compared only with the results presented in AutoML, with SageMaker demonstrating the best performance, followed by Vertex AI and Azure.

Comparatively, the LSTM algorithm demonstrated slightly superior performance within the same environment, although the discrepancy was not statistically significant. These findings align with previous research suggesting comparable performance between LSTM and GRU in various tasks [21]. Nonetheless, the disparity in performance between the two RNN variants appears less pronounced than indicated in existing literature, thus warranting further investigation in this domain.

AWS SageMaker utilizing XGBoost exhibited notably higher accuracy (99.6%), precision (99.8%), recall (99.2%), F1-score (99.5%), and lower log loss (0.014). Such results suggest that this particular algorithm–platform combination may prove more effective than RNNs in specific contexts. These outcomes contrast with the findings of Li et al., who reported RNNs’ superiority over XGBoost in time-series prediction tasks [22]. The divergence could be attributed to differing machine learning model configurations or dataset characteristics employed in the respective studies.

On the other hand, Azure’s LightGBM algorithm displayed inferior performance, yielding an accuracy of 84.20%, precision of 82.20%, recall of 81.80%, F1-score of 81.50%, and log loss of 1.176. Despite underperforming in this study, previous research has indicated LightGBM’s proficiency in handling large datasets with high dimensionality [23].

A noteworthy aspect of our study lies in its comprehensive cross-platform comparison of various machine learning algorithms. Such multi-platform assessments have been infrequently conducted, with most previous studies concentrating on a singular platform or algorithm [24]. Consequently, our research fills a significant void in the field, providing valuable insights to both researchers and practitioners.

Notwithstanding, our study does possess certain limitations. Generalization of the findings to other datasets or tasks might be limited. Thus, future research should encompass diverse datasets and machine learning tasks to validate and extend our discoveries [25]. The notable impact of our study on the field of health and physical education deserves mention. By critically evaluating these algorithms, we contribute to more precise and efficient data analyses, thereby enhancing the comprehension and prediction of health and physical activities [2].

AutoML is an approach to automatically designing and optimizing machine learning models. It automates a series of steps in machine learning, such as data preprocessing, feature selection, and hyperparameter tuning, which could reduce developer workload and improve model performance. However, AutoML still has some limitations. Despite ongoing challenges regarding the limited problem space, AutoML endeavors to discover the optimal solution for a given problem by extensively exploring the space of machine learning [26]. However, the application of AutoML to data on human behavior entails an expansive problem space, making it infeasible or highly inefficient to exhaustively traverse. Hence, AutoML adopts strategies to effectively explore a subset of the problem space, ensuring efficiency. Regarding the constraint on automated decision-making processes, AutoML employs diverse techniques and algorithms to identify the most suitable model. Nonetheless, these decisions necessitate a profound understanding and intuition concerning the efficacy of various techniques [27]. Consequently, AutoML frequently relies on subjective judgment, a factor that poses challenges for complete automation.

In conclusion, this research offers a comparative evaluation of several machine learning algorithms, facilitating a deeper understanding of their respective strengths and weaknesses. Our findings can guide practitioners in selecting the most suitable algorithm for their specific requirements, thereby optimizing the efficacy of their machine learning applications [28].

## 6. Conclusions

This research endeavor involved a meticulous comparative analysis of diverse machine learning algorithms, employing a comprehensive array of performance metrics encompassing accuracy, precision, recall, F1-score, and log loss. Through the rigorous evaluation process, XGBoost, expertly deployed on the esteemed AWS SageMaker platform, emerged as the paramount model, distinctly surpassing its counterparts, including GRU, LSTM, RNN, and LightGBM. The distinguished XGBoost model exhibited a remarkable accuracy of 99.6%, a testament to its robust predictive capabilities.

Furthermore, XGBoost attained the highest rankings in the fundamental aspects of precision, recall, and F1-score, with particular emphasis on the latter’s pertinence in addressing challenges associated with imbalanced data distributions. Of notable significance, the log loss measure, which encapsulates the prediction probabilities, corroborated the finesse of XGBoost’s performance, reflecting an exceptionally low value of 0.014. Consequently, this scholarly investigation culminates in the unambiguous assertion that XGBoost, when thoughtfully harnessed within the distinguished confines of AWS SageMaker, unequivocally manifests itself as the preeminent model, as per the comprehensive metrics considered. However, mindful of the specific nature of the dataset upon which the comparison was founded, the exigency for further inquiry persists, aiming to validate and extend these empirical findings within diverse datasets and real-world contexts.

## Figures and Tables

**Table 1:** Comparison of posture estimation deep learning studies with RNNs ML with our AutoML of this procedure.

		Accuracy (%)	Precision (%)	Recall (%)	F1-Score (%)	Log Loss	Algorithm
RNNs ML	Colab Pro	72.95	70.46	69.28	68.57	0.85	RNN
		73.75	74.55	73.68	73.11	0.71	LSTM
		73.26	74.55	73.33	73.18	0.74	GRU
Auto ML	AWS SageMaker	99.6	99.8	99.2	99.5	0.014	XGBoost
	GCP Vertex AI	89.90	94.20	88.40	91.20	0.268	hidden
	MS Azure	84.20	82.20	81.80	81.50	1.176	LightGBM
	Average Score	91.23	92.07	89.80	90.73	0.49	

AWS: Amazon Web Service, GCP: Google Cloud Platform, MS: Microsoft.

## Data Availability

Data available from the corresponding author S.H. on request.

## References

[1] Diaz S., Stephenson J.B., Labrador M.A. (2019). Use of wearable sensor technology in gait, balance, and range of motion analysis. Appl. Sci..

[2] Choi W., Heo S. (2021). Deep learning approaches to automated video classification of upper limb tension test. Healthcare.

[3] GochOO M., Kim T., Bae J., Kim D., Kim Y., Cho J. (2021). Stochastic Remote Sensing Event Classification over Adaptive Posture Estimation via Multifused Data and Deep Belief Network. Remote Sens..

[4] Liu W., Huang P.-W., Liao W.-C., Chuang W.-H., Wang P.-C., Wang C.-C., Lai K.-L., Chen Y.-S., Wu C.-H. (2022). Vision-Based Estimation of MDS-UPDRS Scores for Quantifying Parkinson’s Disease Tremor Severity. Med. Image Anal..

[5] Dubey S., Dixit M. (2023). A Comprehensive Survey on Human Pose Estimation Approaches. Multimed. Syst..

[6] Chung J.-L., Ong L.-Y., Leow M.-C. (2022). Comparative Analysis of Skeleton-Based Human Pose Estimation. Future Internet.

[7] Garg S., Saxena A., Gupta R. (2022). Yoga Pose Classification: A CNN and MediaPipe Inspired Deep Learning Approach for Real-World Application. J. Ambient Intell. Humaniz. Comput..

[8] Liu A.-L., Chu W.-T. A Posture Evaluation System for Fitness Videos Based on Recurrent Neural Network. Proceedings of the 2020 International Symposium on Computer, Consumer and Control (IS3C).

[9] Yang Y., Wang X., Liu Y., Huang D., Yang Q. (2020). Estimate of Head Posture Based on Coordinate Transformation with MP-MTM-LSTM Network. Int. J. Pattern Recognit. Artif. Intell..

[10] Lin C.-B., Lin Y.-H., Huang P.-J., Wu Y.-C., Liu K.-H. (2020). A Framework for Fall Detection Based on OpenPose Skeleton and LSTM/GRU Models. Appl. Sci..

[11] Sorokina V., Ablameyko S. Extraction of Human Body Parts in Image Using Convolutional Neural Network and Attention Model. Proceedings of the 15th International Conference.

[12] Lai D.K.-H., Yu H., Hung C.-K., Lo K.-L., Tang F.-H.K., Ho K.-C., Leung C.K.-Y. (2023). Dual Ultra-Wideband (UWB) Radar-Based Sleep Posture Recognition System: Towards Ubiquitous Sleep Monitoring. Eng. Regener..

[13] Waring J., Lindvall C., Umeton R. (2020). Automated machine learning: Review of the state-of-the-art and opportunities for healthcare. Artif. Intell. Med..

[14] Wan K.W., Wong C.H., Ip H.F., Fan D., Yuen P.L., Fong H.Y., Ying M. (2021). Evaluation of the performance of traditional machine learning algorithms, convolutional neural network and AutoML Vision in ultrasound breast lesions classification: A comparative study. Quant Imaging Med. Surg..

[15] Siriborvornratanakul T. (2022). Human behavior in image-based Road Health Inspection Systems despite the emerging AutoML. J. Big Data.

[16] AIHub by the Korea Intelligence Society Agency (2023). https://aihub.or.kr/aihubdata/data/list.do?pageIndex=1&currMenu=115&topMenu=100&da-ta-SetSn=&srchOrder=&SrchdataClCode=DATACL001&searchKeyword=&srchDataRealmCode=REALM006.

[17] Amazon Web Services (2021). Build, Tune, and Deploy an End-to-End Churn Prediction Model Using Amazon SageMaker Pipelines. https://aws.amazon.com/ko/blogs/machine-learning/build-tune-and-deploy-an-end-to-end-churn-prediction-model-using-amazon-sagemaker-pipelines/.

[18] (2021). Google Cloud. End-to-End AutoML Workflow. https://cloud.google.com/vertex-ai/docs/tabular-data/tabular-workflows/e2e-AutoML?hl=ko#end-to-end_on.

[19] Microsoft (2021). Azure Machine Learning Solution Architecture. https://learn.microsoft.com/en-us/azure/architecture/solution-ideas/articles/azure-machine-learning-solution-architecture.

[20] Zhu F., Hua W., Zhang Y. (2023). GRU Deep Residual Network for Time Series Classification. Proceedings of the 2023 IEEE 6th Information Technology, Networking, Electronic and Automation Control Conference (ITNEC).

[21] Chung J., Gulcehre C., Cho K., Bengio Y. Empirical Evaluation of Gated Recurrent Neural Networks on Sequence Modeling. Proceedings of the Neural Information Processing Systems, NIPS.

[22] Li S., Mingyu S. (2022). A comparison between linear regression, lasso regression, decision tree, XGBoost, and RNN for asset price strategies. Proceedings of the International Conference on Cyber Security, Artificial Intelligence, and Digital Economy (CSAIDE 2022).

[23] Pan H., Li Z., Tian C., Wang L., Fu Y., Qin X., Liu F. (2023). The LightGBM-based classification algorithm for Chinese characters speech imagery BCI system. Cogn. Neurodyn..

[24] Garcia S., Luengo J., Herrera F. (2015). Data Preprocessing in Data Mining.

[25] Bishop C.M., Nasrabadi N.M. (2006). Pattern Recognition and Machine Learning.

[26] He X., Zhao K., Chu X. (2019). Automl: A Survey of the State of-the-Art. arXiv.

[27] Zaharia S., Rebedea T., Trausan-Matu S. (2022). Machine Learning-Based Security Pattern Recognition Techniques for Code Developers. Appl. Sci..

[28] Dong X., Yu Z., Cao W., Shi Y., Ma Q. (2020). A survey on ensemble learning. Front. Comput. Sci..

